# Novel﻿ eco-friendly low cost and energy efficient synthesis of (Nd–Pr–Dy)_2_Fe_14_B magnetic powder from monazite concentrate

**DOI:** 10.1038/s41598-021-99464-w

**Published:** 2021-10-18

**Authors:** Syed Kamran Haider, Jin-Young Lee, Amol Uttam Pawar, Dongsoo Kim, Young Soo Kang

**Affiliations:** 1 Convergence Research Centre for Development of Mineral Resources, Korea Institute of Geoscience and Mineral Resources, 124, Gwahakro, Yuseonggu, Daejeon, 34132 Korea; 2 Powder and Ceramics Division, Korea Institute of Materials Science, 797, Changwondaero, Seongsangu, Changwon, Gyeongnam, 51508 Korea; 3 Department of Chemistry, Sogang University, 35, Baekbeomro, Mapogu, Seoul, 04107 Korea

**Keywords:** Materials science, Chemistry, Chemical engineering, Green chemistry, Materials chemistry, Process chemistry

## Abstract

Syntheses of Nd_2_Fe_14_B magnetic powder by conventional method is a complicated multi-step process, which produces harmful pollutants and consumes a huge amount of energy and resources. Herein we report a simple chemical route for the preparation of (Nd–Pr)_2_Fe_14_B magnetic powder using monazite concentrate as a precursor. Th, U, Sm, and La impurities were removed from monazite leachate by roasting, solvent extraction and leaching the concentrate. Purified leachate consisting of Nd and Pr Chlorides was added to the FeCl_3_ solution, and the solution produced was co-precipitated with NaOH. RE and Fe hydroxide precipitates were converted to the oxides by annealing at 700 °C. Boric acid and CaH_2_ were added in the RE and Fe oxides produced, and this mixture was reduced and diffused to (Nd–Pr)_2_Fe_14_B. Magnetic properties of the (Nd–Pr)_2_Fe_14_B produced were enhanced by introducing antiferromagnetic coupling, induced by Dy addition and efficient removal of CaO byproduct through ball milling in ethanol which increased the BH_max_ from 3.9 to 11.45 MGOe. Process reported is energy efficient, environment-friendly, time saving and low-cost.

## Introduction

Rare earth elements (RE) have a wide variety of applications in high-tech products and permanent magnets, hence their demand is increasing rapidly. Monazite is a rich source of various RE, since 70% of the monazite consists of the oxides and phosphates of various RE, mainly of Ce, La, Nd, Pr, and Sm^1–7^. Crystalline phosphate ore in monazite has high chemical and thermal stability, therefore it is quite difficult to extract RE from it. Generally, two chemical methods (acid digestion and leaching process) are commercially applied to obtain the RE from monazite. These processes require high temperature (230 °C) and sulfuric acid to roast the concentrate. Roasting is followed by digestion in which a large quantity of inorganic bases (e.g. NH_4_OH, NaOH) hydroxide are used^8,9^. After roasting, digestion, multiple solvent extractions, leaching, and precipitation, RE-oxides are obtained^10^. RE-oxides are reduced to the RE metals by molten salt electrolysis. Reduced RE's are alloyed to the (Nd-RE)_2_Fe_14_B (RE = Nd, Dy, etc.) after addition of Fe and B. Two major commercialized routes for the production of Nd_2_Fe_14_B magnetic powder are powder metallurgy and melt spinning process. Both of these processes are complicated and need high temperature for alloying steps (e.g. induction melting, strip casting. crushing, Jet milling). Conclusively it takes long time, huge amount of energy and resources to obtain pure RE metals from the monazite and then synthesis of Nd_2_Fe_14_B magnetic powder from them. Among permanent magnets, Nd_2_Fe_14_B hard magnets exhibit the highest magnetic properties such as magnetic remanence and energy density^11–18^. Hence Nd_2_Fe_14_B magnets have huge expanding a market, and it is important to introduce a low-cost, eco-friendly, energy-efficient and time saving method for their production (Fig. S-1).

Herein we report a novel, simple, and convenient chemical method to synthesize (Nd–Pr)_2_Fe_14_B (RE = Nd and Pr) from monazite concentrate. Monazite concentrate was refined by roasting, solvent extraction, and leaching in HCl. Obtained leachate was mixed with FeCl_3_, then mixture was co-precipitated (to produce RE and Fe hydroxides) and annealed to produce RE and Fe oxides. Boric acid and CaH_2_ were added in the RE and Fe oxides and metal oxides were converted to the (Nd–Pr)_2_Fe_14_B by reduction-diffusion (R-D) process. Magnetic properties of the (Nd–Pr)_2_Fe_14_B produced are improved by Dy addition and (removal of CaO through) ball milling in ethanol.

## Experimental section

### Materials

Iron (III) chloride hexahydrate (FeCl_3_·6H_2_O), sodium hydroxide (NaOH), boric acid (H_3_BO_3_), calcium hydride (CaH_2_), dysprosium (III) chloride hexahydrate (DyCl_3_·6H_2_O), ethyl alcohol (C_2_H_5_OH), sulfuric acid (H_2_SO_4_), hexane (C_6_H_6_) and acetone (CH_3_COCH_3_) were obtained from Sigma-Aldrich Co.

Primene JM-T and Cyanex 572 were received from Guide chem and CYTEC industries, respectively. All chemicals used in this work were of analytical grade. Korean monazite mineral was provided by convergence research center for development of mineral resources, Korea Institute of geoscience and mineral resources. Visible impurities were removed from the monazite mineral without using any chemical and purified mineral was named monazite concentrate which used as a precursor of Nd and Pr.

### Characterization

The concentration of the elements in the monazite leachate was determined by inductively coupled plasma atomic emission spectrometry (ICP-AES, Shimadzu). The morphology, size, and elemental distribution of the intermediate and final product particles were observed with scanning electron microscopy (SEM, MERLIN) and transmission electron microscopy (JEM-2100F TEM, JEOL) operated at 200 kV embedded with energy-dispersive X-ray spectroscopy (EDS). Crystal structure analysis was done with X-ray diffractometer (Rigaku MiniFlex) with Cu-Kα source, with radiation wavelength of 0.15418 nm. For intermediate products, TEM, TEM-EDS, and HRTEM characterizations were performed on JEM-2100F by JEOL Ltd. For TEM analysis, RE and Fe oxide particles were dispersed in ethanol. Magnetic properties were measured by physical property measurement system (PPMS, Evercool II–9T) in the vibrating sample magnetometer mode. JEOL JEM-ARM200F Cs-corrected TEM was used to obtain “High-angle annular dark-field imaging scanning transmission electron microscopy” (HAADF-STEM) and “Low-angle annular dark-field imaging scanning transmission electron microscopy” (LAADF-STEM) images.

### Experimental process

**Figure 1:**
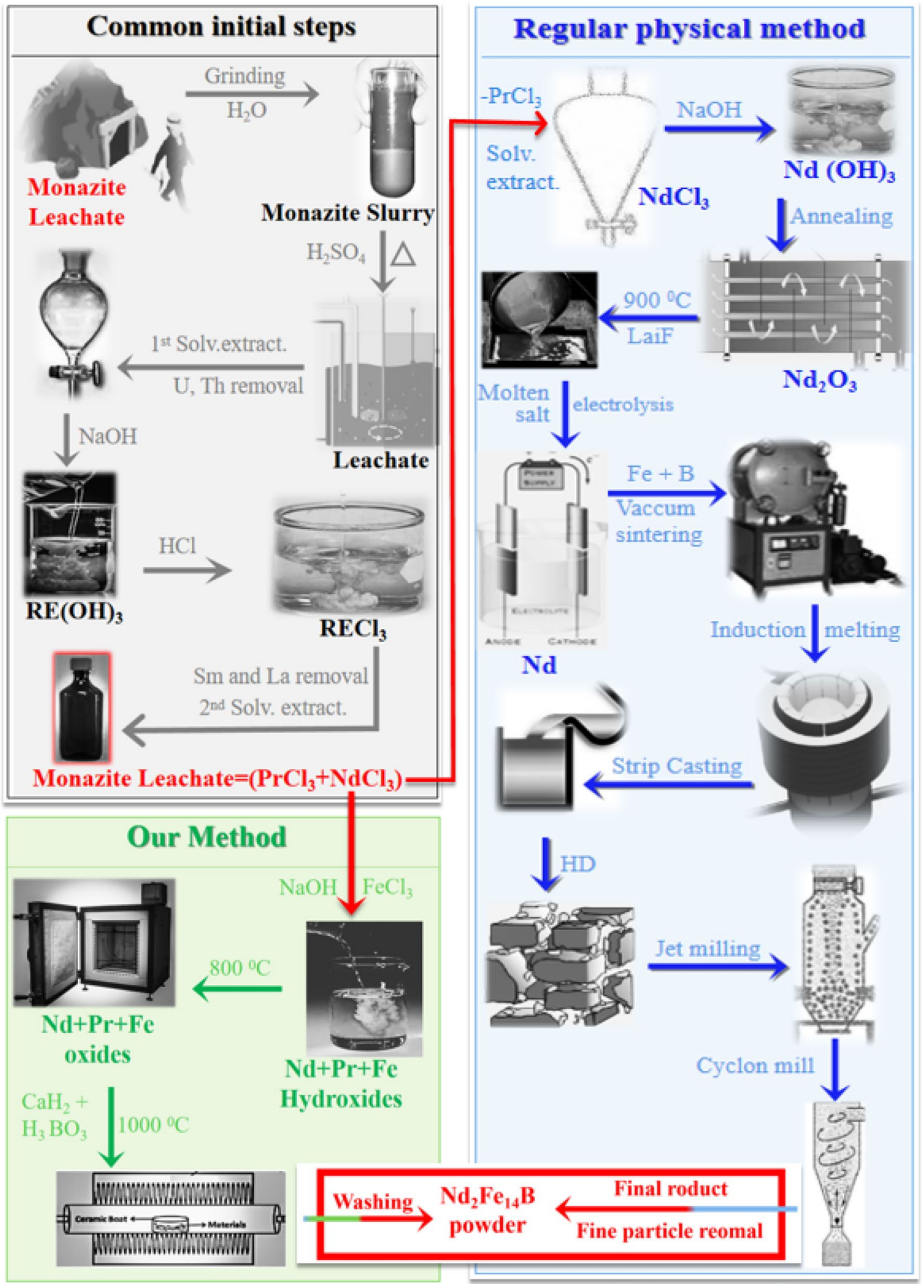
(**a**) Flow chart of our experimental process compared with the regular commercial process.

Our study is mainly reporting an experimental process; hence the results and discussion section will also explain the experimental process in detail. To avoid the repetition of the information, experimental process has been moved to the supporting information. However, a flow chart of the experimental process is provided as Fig. 1.

## Results and discussion

Monazite concentrate used in this work mainly consisted of phosphates and oxides of RE, Th, and U. At first, the concentrate was ground and sieved, as particle size approached 44–500 μm. Almost half of the sample passed through 150 mesh (105 μm) sieve. Sample was mixed with water in such a way that slurry with density of a 100 g/L was formed. The first step was the removal of Th and U from monazite by roasting in (6 N) H_2_SO_4_. Concentration of both the Th and U was recorded as ~ 0.42 g/L in the slurry. Efficiency of the process was determined by various factors e.g., leaching time, leaching temperature and acid to slurry ratio. Experimental conditions were changed in various experiments and optimum conditions were determined. In optimized process, acid to slurry weight ratio was kept as 1:3 and slurry was roasted at 220 °C for 90 min. Following equation explains the chemical reactions those took place during the roasting step.

$$\begin{aligned} & {\text{REPO}}_{4} + \, 3{\text{H}}_{2} {\text{SO}}_{4} \to {\text{RE}}_{2} \left( {{\text{SO}}_{4} } \right)_{3} + \, 3{\text{H}}_{3} {\text{PO}}_{4} \\ & {\text{Th}}_{3} \left( {{\text{PO}}_{4} } \right)_{4} + \, 6{\text{H}}_{2} {\text{SO}}_{4} \to 3{\text{Th}}\left( {{\text{SO}}_{4} } \right)_{2} + \, 4{\text{H}}_{3} {\text{PO}}_{4} \\ & {\text{U}}_{3} \left( {{\text{PO}}_{4} } \right)_{4} + \, 6{\text{H}}_{2} {\text{SO}}_{4} \to 3{\text{U}}\left( {{\text{SO}}_{4} } \right)_{2} + \, 4{\text{H}}_{3} {\text{PO}}_{4} \\ \end{aligned}$$

To separate U and Th in the form of sulfate salts, Primene JM-T was used. Detail of the solvent extraction process is described in the supporting information.
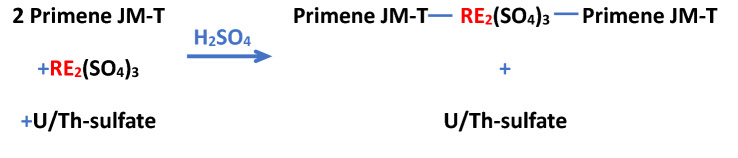


Primene JM-T is a popular reagent used for the solvent extraction of RE. Molecules of the Primene JM-T make organometallic complex with the RE sulfate selectively^19^. During the solvent extraction, sulfates of both the U and Th remained dissolved in the aqueous phase and were separated.
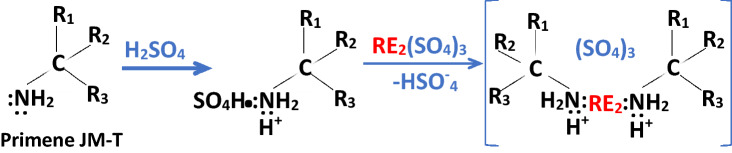


After the extraction of Th and U, in the next step, La and Sm were removed. Solution obtained from the first step was precipitated with (3 M) NaOH until pH value approached 10. RE(OH)_3_ produced were leached in HCl, hence leachate containing La (12.84 g/L), Pr (1.720 g/L), Nd (5.35 g/L), and Sm (less than 0.01 g/L) was obtained. Sm and La were removed by solvent extraction with Cyanex-572. Detail of the solvent extraction process is described in the supporting information.
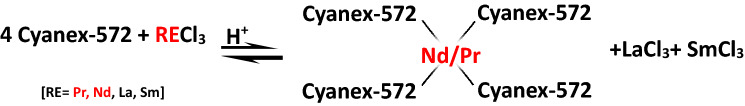


Cyanex-572 is mixture of phosphinic and phosphonic acids^20^, which dimerizes and preferentially separates the Nd and Pr from Sm and La. By using Cyanex-572, almost 99.94% of the Sm and La were removed from the leachate.
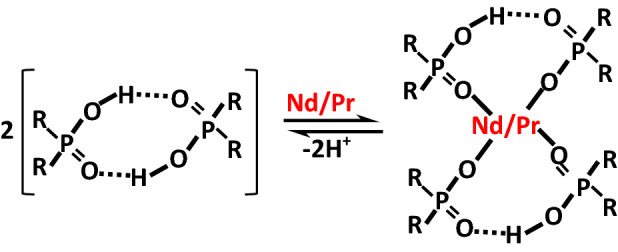


It was evaluated by ICP-AES analysis that the solution obtained after second solvent extraction, consisted of mixtures of chlorides of various RE (Table 1). This solution was named as monazite leachate. Although leachate consisted of 16 different RE but more than 99% of the leachate comprised of Nd and Pr. Nd:Pr ratio was recorded as 3.83:1.

**Table 1:** Elemental composition of the monazite leachate determined by the ICP-AES analysis.

Elements	Concentration (ppm)
Y	< 1
La	< 1
Ce	< 1
Pr	11,070
Nd	42,480
Sm	174.4
Eu	77.78
Tb	< 1
Dy	70.36
Ho	8.835
Er	< 1
Tm	2.798
Yb	2.622
Lu	< 1
Th	< 1
U	< 1
Gd	212.4

FeCl_3_ was added in the monazite leachate solution in such a way that RE:Fe (RE = Nd + Pr) ratio was kept as 15:77. For the synthesis of (Nd–Pr)_2_Fe_14_B by R-D, physical contact between constituent elements in the precursor is very important, because it leads to efficient solid-state diffusion. Hence in this work, co-precipitation was employed for the synthesis of oxide powder. Initial pH of the monazite leachate was 2.64. As NaOH solution was added, red precipitates of Fe (OH)_3_ were produced and dissolved again immediately. pH of the solution changed slowly until 4 and then after adding very little NaOH, it suddenly raised to the 7.

All RE and Fe Chlorides were converted to hydroxides as presented in the equations below:

$$\begin{aligned} & {\text{FeCl}}_{3} + 3{\text{NaOH }} \to {\text{Fe}}\left( {{\text{OH}}} \right)_{3} + 3{\text{NaCl}} \\ & {\text{RECl}}_{3} + 3{\text{NaOH}} \to {\text{RE}}\left( {{\text{OH}}} \right)_{3} + 3{\text{NaCl}} \\ \end{aligned}$$

Monazite leachate was co-precipitated at pH 7, 8, 9, 10, 11, 12, and 13 in separate experiments. After co-precipitation at pH 7, 8, 9 and 10, some of the precipitates were suspended in the solution and it took more than 2 h for them to settle down (Fig. S-7). But after co-precipitation at pH 13, it took 25 min for precipitates to settled down (Fig. S-7) completely. When precipitates settled down completely, more than 80% of the by-product (solution of the NaCl and NaOH dissolved in the solution) can be removed just by decanting, without any centrifugation. Hence for the co-precipitation, pH 13 was selected as the optimum pH. In order to remove the co-precipitation by-products (NaCl and NaOH) completely, centrifugation at 4000 rpm for 45 s was carried out.

**Figure 2:**
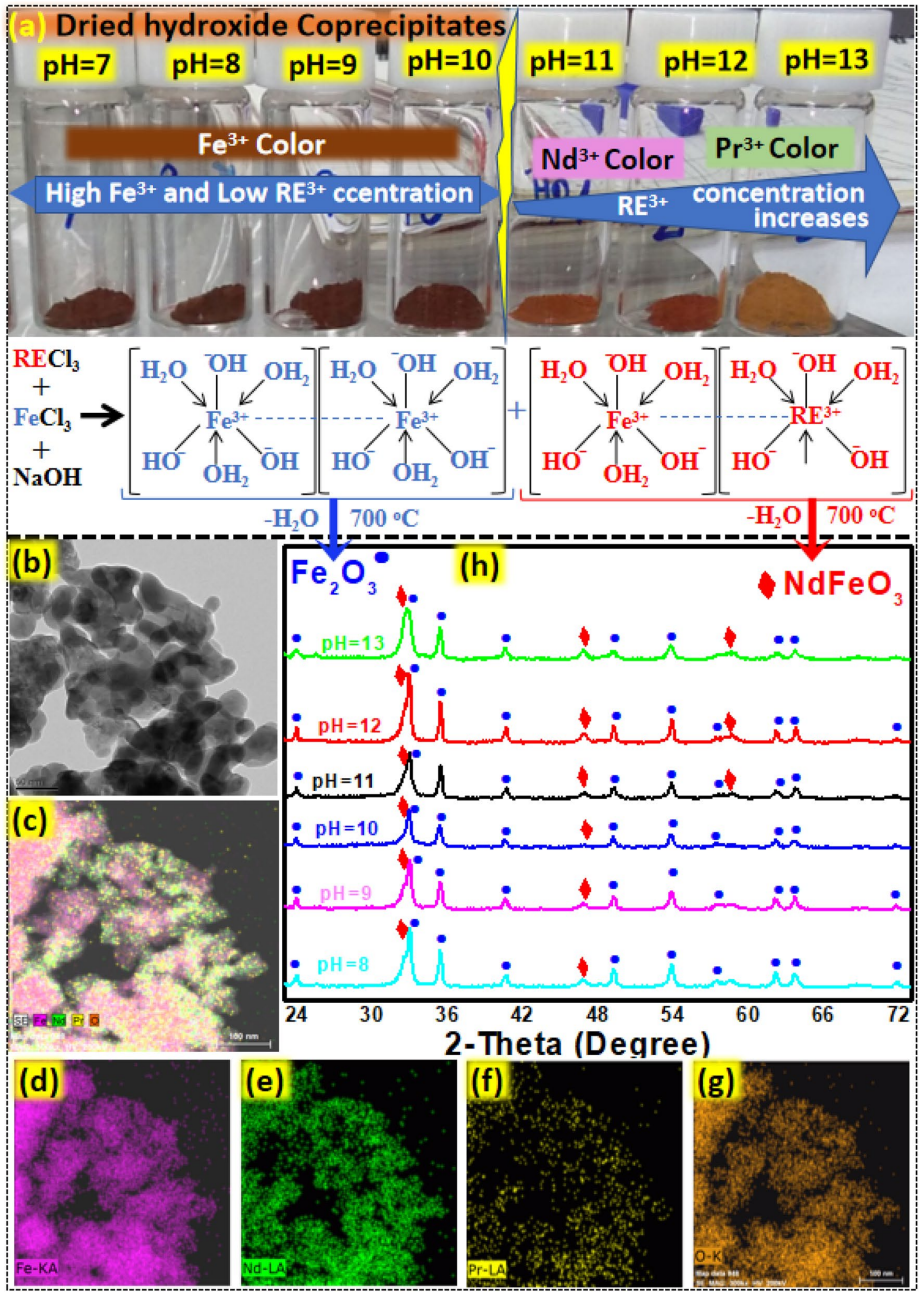
(**a**) Color of the dried hydroxides precipitates precipitated at pH 7–13 (**b**–**g**) TEM and TEM-EDS images of the RE and Fe oxide particles produced at pH 13. (h) XRD patterns for oxides of RE and Fe, produced by annealing their hydroxides.

Fe^3+^ forms highly acidic complexes in monazite leachate. After the addition of NaOH, Fe forms aqua complexes [Fe(H_2_O)_3_(OH)_3_]. [Fe(H_2_O)_3_(OH)_3_] is not stable at pH below 7, however, when pH exceeds 7, neutral complexes of Fe precipitate out forming hydroxides. This is the reason that percentage yield increases with increasing the pH. Similarly, RECl_3_ is converted to [RE(H_2_O)_3_(OH)_3_]. By increasing pH more Nd and Pr were precipitated. Colors of Fe^3+^, Nd^3+^, and Pr^3+^ are brick red, pink, and light green, respectively. Figure 2a show that precipitation at higher pH produced more [RE(H_2_O)_3_(OH)_3_] as the color of the hydroxide mixture slightly turns brown. Hydroxides of both Fe and RE produced by co-precipitation were amorphous and could not be detected in XRD. [RE(H_2_O)_3_(OH)_3_] and [Fe(H_2_O)_3_(OH)_3_] were converted to the REFeO_3_ (mainly (NdFeO_3_)) and Fe_2_O_3_ by annealing at 700 °C in the presence of air. Oxides produced at various pH were characterized by TEM (Fig. 2 b–g) and XRD (Fig. 2h). By increasing the pH, precipitation of the RE enhanced which could be easily detected around 33 degree in XRD patterns. Precipitation at higher pH reduces the Fe_2_O_3_ peaks at various points on the XRD patterns, which is more prominent at 72° of 2-theta, where Fe_2_O_3_ peaks diminished by precipitation at higher at higher pH. Reduction in the Fe_2_O_3_ peaks and formation of more REFeO_3_ indicate the increased precipitation of RE at higher pH. TEM analysis revealed that Fe_2_O_3_ and NdFeO_3_ had an average particle size of 35 nm (Fig. 2b). TEM–EDX analysis shows the distribution of the RE and Fe oxide particles. (Fig. 2c–g)). Size distribution of the oxide particles is provided in the Fig. S-9.

Co-precipitation at pH 13 gave the highest percentage yield (~ 99%) of RE and Fe oxides (Fig. S-10), hence oxides produced only at pH 13 were processed further for the next step, R-D. Oxide mixture was mixed with boric acid and CaH_2_ and then pressed into pellet form. Pellet was annealed at 1000 °C in the inert environment for the complete R-D of the oxides.

In a separate experiment, DyCl_3_.6H_2_O was added in monazite solution before co-precipitation. The rest of the experiment was performed the same as the previous experiment. Dy content consequently enhanced the magnetic properties of the final product. Product obtained in this experiment RE_2_Fe_14_B will is labeled as (Nd–Pr)_1.5_Dy_0.5_Fe_14_B.

During the R-D, CaO and H_2_ were produced as by-product. Produced CaO is helpful to control the particle size by Ostwald ripening during the diffusion and stops the formation of oversized (Nd–Pr)_2_Fe_14_B particles^21^. However, being non-magnetic CaO byproduct reduces the magnetic properties of the magnetic particles. (Nd–Pr)_2_Fe_14_B and, (Nd–Pr)_1.5_Dy_0.5_Fe_14_B were washed with water to remove the CaO. But water reacted with the CaO and formed Ca(OH)_2_ which is sparingly soluble in water. Consequently, this non-magnetic Ca(OH)_2_ was left in the (Nd–Pr)_2_Fe_14_B after washing.

For the more efficient removal of the CaO, in another experiment, (Nd–Pr)_1.5_Dy_0.5_Fe_14_B was ball milled in ethanol. CaO removal by ethanol ball milling in this work is different from the study that we reported earlier^22^. In our previous study, milling was performed in a planetary ball milling machine at 300 rpm for 3 h which was quite useful but still had few disadvantages. Milling in the close vessel at high rpm for 3 h increased the temperature of the vessel, which may lead to the partial oxidation of the product. Hence a separate cooling system was attached with the milling apparatus. Secondly, planetary ball milling cannot be used at industrial scale as efficiently as table ball milling. Furthermore, milling at intense conditions as above contaminates the balls and bowl of the milling apparatus and it is not convenient to wash the apparatus after every washing. Hence, in this study common table ball milling apparatus with glass vial (as milling jar) was used, which is much cheaper as compared to the Pulverisette-6 planetary ball mill machine, that was used in the previous study. Most importantly it was found that that table ball mill removed CaO as effectively as the planetary ball mill.

In the current study, 1.5 g of R-D product ((Nd–Pr)_1.5_Dy_0.5_Fe_14_B+ CaO) was ground and dispersed in the ethanol (20 ml) in a glass vial. Zirconia balls (70 g) were added to the above mixture and the vial was sealed with the Teflon tap after closing the lid. Ball milling was performed for 3 h at 180 rpm in the open air and then the product obtained was washed with the water thrice to separate the non-magnetic by-products. After the water washing, product was rinsed with acetone twice then stored in the glove box in the inert environment. Final product obtained in this experiment was labeled as (Nd–Pr)_1.5_Dy_0.5_Fe_14_B (BM).

**Figure 3:**
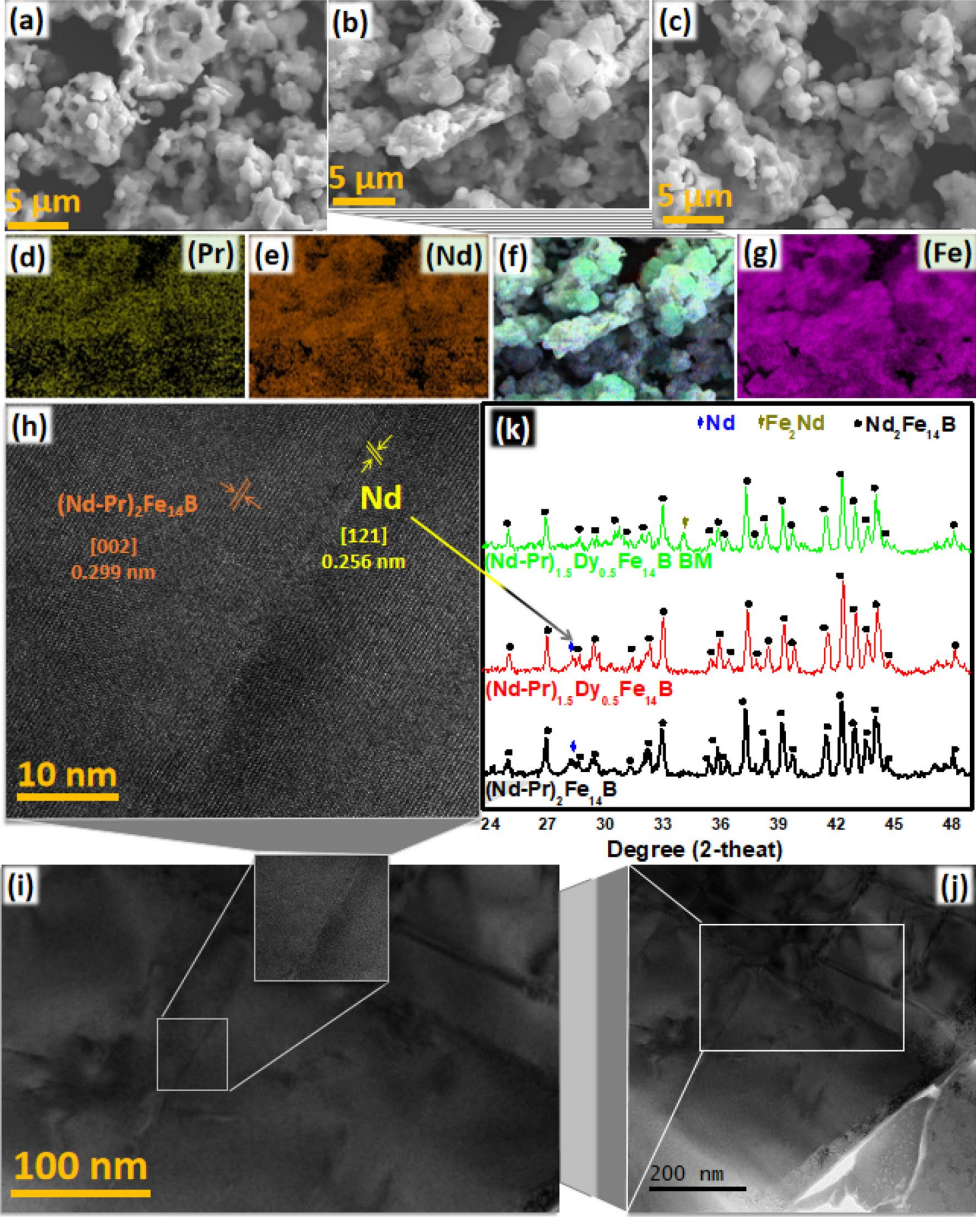
SEM images of (**a**) (Nd–Pr)_1.5_Dy_0.5_Fe_14_B (b) (Nd–Pr)_2_Fe_14_B (**c**) (Nd–Pr)_1.5_Dy_0.5_Fe_14_B (BM). (**d**–**g**) SEM–EDS of images of (Nd–Pr)_2_Fe_14_B (**h**) HAADF-STEM image of (Nd–Pr)_2_Fe_14_B (**i**–**j**) LAADF-STEM image of (Nd–Pr)_2_Fe_14_B B (**k**) XRD patterns for all three products.

Detailed characterization of all three products, (Nd–Pr)_2_Fe_14_B, (Nd–Pr)_1.5_Dy_0.5_Fe_14_B, and (Nd–Pr)_1.5_Dy_0.5_Fe_14_B (BM) was performed. XRD patterns of all three products described above are very similar. This is because of their very similar crystal structures. However, the ethanol ball milled product did not show any Nd peak (Fig. 3k). Most probably non-magnetic RE phase attached with the magnetic product was removed during ball milling process. From SEM images, average particle size of the (Nd–Pr)_2_Fe_14_B, (Nd–Pr)_1.5_Dy_0.5_Fe_14_B and (Nd–Pr)_1.5_Dy_0.5_Fe_14_B (BM) was estimated as 2, 1.5 and 1.3 μm (Fig. 3a–c). SEM–EDS shows the elemental distribution of RE and Fe (Fig. 3d–g) in (Nd–Pr)_1.5_Fe_14_B. SEM–EDS images provided in the supporting information show the elemental distribution in all three products (Fig. S-11,12,13). This further demonstrates that how after ball milling in ethanol reduced the Ca proportion (Fig. S-11,12,13).

To study the micro-structure, LAADF-STEM and HAADF-STEM analysis was performed (Fig. 3h–j). HAADF-STEM image of (Nd–Pr)_2_Fe_14_B shows two mixed crystalline lattice fringes, one is related to (002) plane of (Nd–Pr)_2_Fe_14_B with the d-spacing value of 0.299 nm and another one is related to (121) plane of Nd phase with d- spacing value 0.256 nm, as shown in Fig. 3h. Further confirmation of the Nd phase was monitored by a typical XRD pattern and SEM–EDS images presented in Fig. 3k,d–g, respectively. Additionally, one extra diffraction peak appeared at 2-theta is equal to 28.2° corresponds to the (121) plane of the Nd phase.

There is no evidence that all (~ 25%) the Dy is substituted in the Nd_2_Fe_14_B crystal lattice. Two main evidence for the Dy substitution in the Nd_2_Fe_14_B come from the XRD and HRTEM. In case of the (~ 25%) Dy substitution in Nd_2_Fe_14_B, XRD peaks are shifted towards right side, because of smaller size of Dy (as compared to the Nd)^21^. In our study, no peak shift was observed and there was no evidence of complete Dy substitution in the HRTEM analysis. However, there is a strong possibility that Dy is partially substituted in the Nd_2_Fe_14_B crystal lattice which is also evident in SEM–EDS. Usually, when Nd_2_Fe_14_B is prepared by the R-D method, added Dy prefers to substitute inside the crystal lattice. However, in our precursors there are 16 RE in the precursors, hence, it is very difficult to know that which RE will substitute inside the lattice and which will remain outside the lattice.

**Figure 4:**
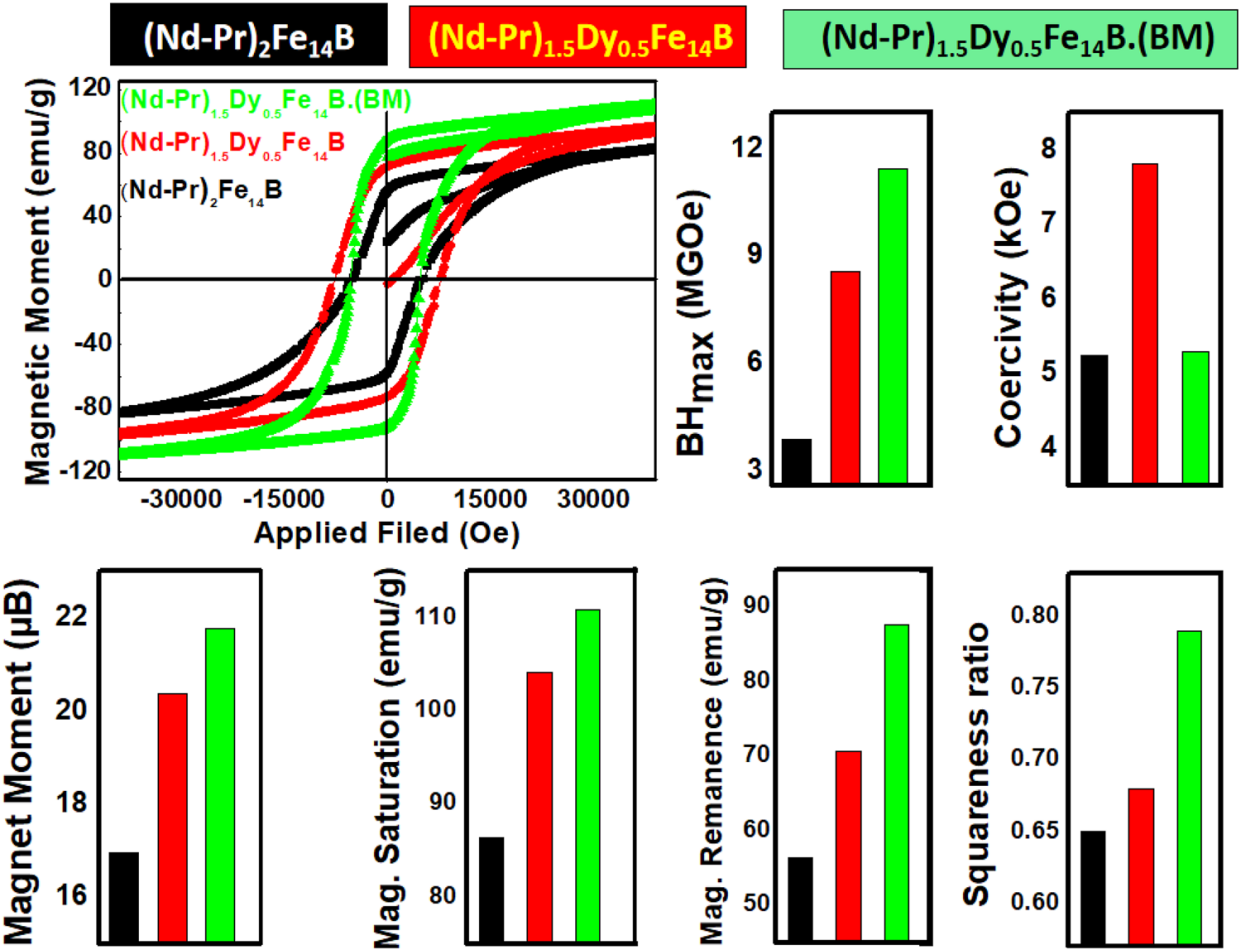
Magnetic hysteresis loop and comparison of magnetic properties of (Nd–Pr)_2_Fe_14_B, (Nd–Pr)_1.5_Dy_0.5_Fe_14_B and (Nd–Pr)_1.5_Dy_0.5_Fe_14_B (BM).

Magnetic properties of all the products together with hysteresis loop are given in Fig. 4. Magnetic moments in (*μ*_B_) are calculated (per formula unit of (Nd–Pr)_2_Fe_14_B from M_s_ values. Experimentally determined values of magnetic moment (*μ*_B_) in this work are lower than the values predicted by theoretical calculation^22^ because experimental conditions conditions are not fixed as theoretical. Theoretical values were calculated assuming the temperature as 5 K (no thermal energy effect), and it was assumed that all particles are single domain and un-oxidized (no washing effect).

Studying the effect of Pr on the of magnetic properties of (Nd–Pr)_2_Fe_14_B is not a straightforward case. Reduction in the Ms and enhancement in the coercivity has been reported after the Pr addition to the Nd_2_Fe_14_B^23,24^. But meanwhile contradictory results have also been observed. Zhang et al^25^ reported the reduction in squareness ratio (enhancement in Ms value) after Pr addition to the Nd_2_Fe_14_B. Imran et al^26^ quantitatively measured the enhancement of the magnetic moment (*μ*_B_/formula unit), when 25% Pr was substituted in the Nd_2_Fe_14_B crystal lattice. They reported that overall magnetic moment was enhanced after the Pr substitution (because of the Nd–Pr ferromagnetic coupling).

Main reason for these contradicting results is the position of Pr, in the (Nd–Pr)_2_Fe_14_B based magnetic material. If the Pr is outside the Nd_2_Fe_14_B crystal and found on the grain boundary, it has negligible effect on the magnetic moment. However, in this case it decouples the Nd_2_Fe_14_B grain boundaries effectively. This decoupling enhances the coercivity (and may also reduces the Ms value). If Ms value is reduced this is mainly because of enhancement in the anisotropy energy and coercivity. However if the Pr is substituted in the (Nd–Pr)_2_Fe_14_B crystal lattice overall magnetic moment of the lattice will increase. Enhanced magnetic moment (Ms value) will reduce anisotropy energy and coercivity. During the synthesis of (Nd–Pr)_2_Fe_14_B by R–D method (we also used the R-D method) Pr is usually substituted inside the Nd_2_Fe_14_B crystal. Furthermore, HAADF-STEM image (Fig. 3h) also agrees with this statement. That is why in our study, Pr substitution enhanced the Ms value, meanwhile, both the anisotropy energy and the coercivity reduced slightly. The role of Pr on the effect of structural and magnetic properties is further explained in the supporting information.

Dy exhibits almost three times higher magnetic moment as compared to the Nd or Pr and it can affect the magnetic moment of (Nd–Pr)_1.5_Dy_0.5_Fe_14_B in two different ways. If Dy substitutes in the Nd_2_Fe_14_B crystal, it couples antiferro-magnetically with the surrounding elements. However, if Dy is found outside the crystal lattice, it is found as Dy rich phase and there is no antiferro-magnetic coupling involved. Furthermore, paramagnetic behaviour of the Dy can enhance the magnetic moment of the final product. Dy might be oxidized during washing process and produced oxide, which acted as soft magnet. Hence, Dy or Dy_2_O_3_ phases enhanced the overall magnetic moment of the magnetic particles.

The response of an ideal, homogeneous paramagnetic particles to applied external magnetic field is given by a Langevin function^27^:

$${\text{M}} = {\text{M}}_{s} {\text{L}}[\mu \mu_{0} H/K_{B} T\left] { = {\text{NL}}} \right[\mu \mu_{0} H/K_{B} T]$$

where M represents the total magnetization, M_s_ is the saturation magnetization, µ is magnetic moment of Dy or Dy_2_O_3_ rich phase, µ_0_ is vacuum permeability constant, H is applied field, k_B_ is Boltzmann’s constant, T is temperature, N is density of magnetically-active atoms in the Dy phase, and L(x) = coth(x) − 1/x^27^. Equation above states that magnetization of the Dy or Dy_2_O_3_ rich phase is directly proportional to the quantity as well as the magnetic moment of the Dy.

**Figure 5:**
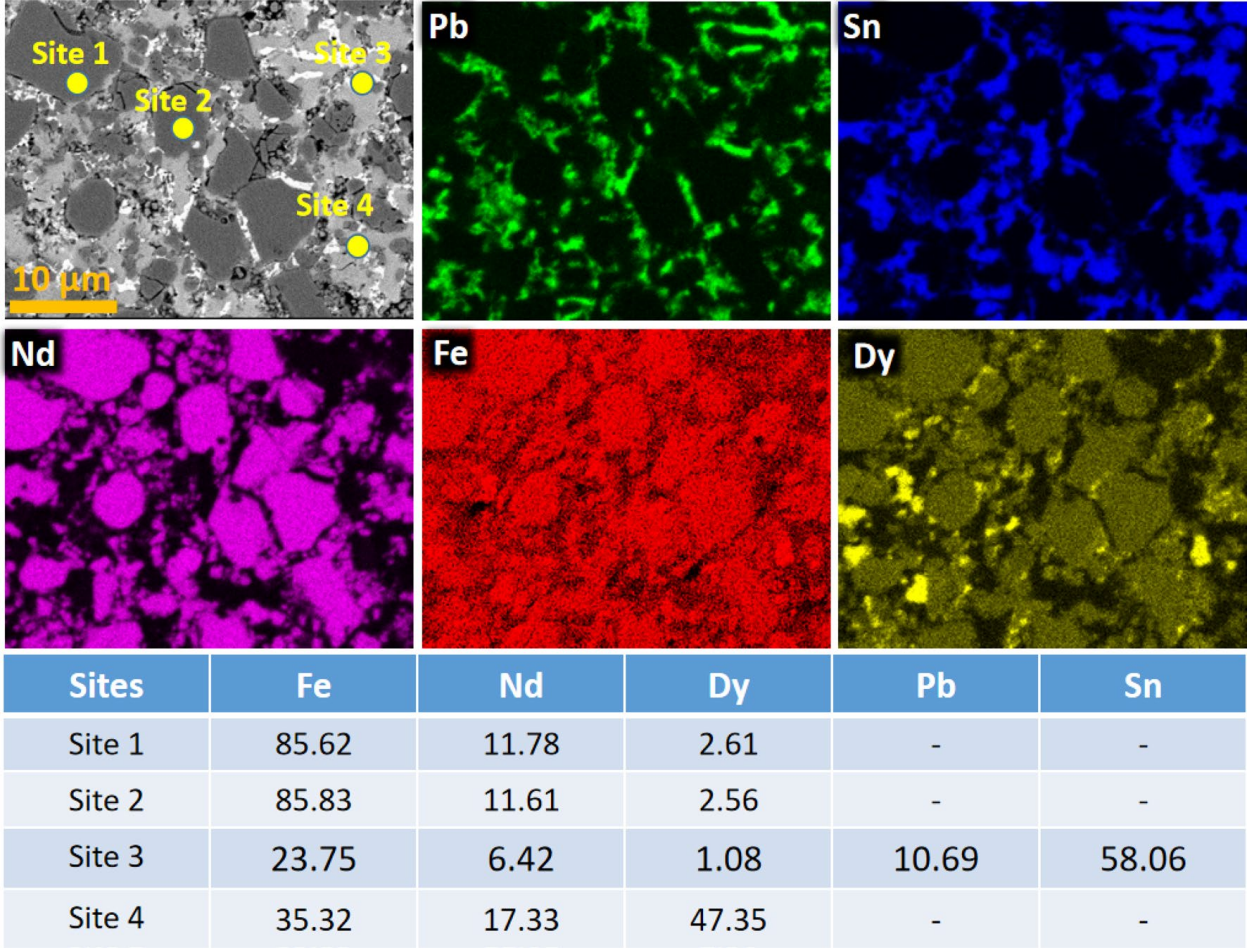
BSE-SEM and BSE-SEM–EDS images of (Nd–Pr)_1.5_Dy_0.5_Fe_14_B.

Hence, it is proposed that when more Dy is substituted inside the crystal lattice magnetic moment of (Nd–Pr)_1.5_Dy_0.5_Fe_14_B will decrease. On contrary, if more Dy is found outside a crystal lattice overall magnetic moment will increase. Enhanced magnetic moment in (Nd–Pr)_1.5_Dy_0.5_Fe_14_B suggests that there is more Dy outside the crystal lattice as compared to the inside. BSE-SEM and BSE-SEM–EDS images of (Nd–Pr)_1.5_Dy_0.5_Fe_14_B further support this statement (Fig. 5). BSE-SEM analysis was performed by the process reported by Hiader et al.^22^ which indicates three regions. One is the main (Nd–Pr)_1.5_Dy_0.5_Fe_14_B phase which is prominent with dark gray color. EDS measurement for site 1 and 2 are taken from that region. Site 3 represents the area which has high concentration of Pb and Sn. These two elements come from the solder. Site 4 indicates the presence of Dy phase, which the Dy phase outside the (Nd–Pr)_1.5_Dy_0.5_Fe_14_B crystal lattice. Conclusively, this Dy increases the magnetic moment of the final product.

Beside the Magnetic Moment Dy addition always increases the anisotropic energy. It is a common observation that when added to Nd_2_Fe_14_B, Dy enhances anisotropy energy, M_r_ and BH_max_^28–33^. In this work, Dy was also added to increase the anisotropy field and BH_max_. BH_max_ of the (Nd–Pr)_1.5_Dy_0.5_Fe_14_B approached to 8.56 MGOe (Fig. 4). CaO produced during R-D process significantly reduces the magnetic properties of the produced magnetic particles^34^. BH_max_ of the (Nd–Pr)_1.5_Dy_0.5_Fe_14_B was improved up to 11.45 MGOe (Fig. 4) by more efficient remal of CaO through ball milling in ethanol.

The addition of Dy increased the coercivity of (Nd–Pr)_1.5_Dy_0.5_Fe_14_B (7.83 kOe) as compared to the (Nd–Pr)_2_Fe_14_B (5.24 kOe). The main reason behind the enhancement in coercivity is the stronger anisotropic field created by Dy. (Nd–Pr)_1.5_Dy_0.5_Fe_14_B had smaller particle size as compared to the (Nd–Pr)_2_Fe_14_B (Fig. 2). It is well-known fact that as particle size gets smaller and approaches the single domain size, coercivity increases. The particle size effect further enhanced the coercivity of (Nd–Pr)_1.5_Dy_0.5_Fe_14_B. Another important factor that affected the coercivity was Ca(OH)_2_ which contaminated the final product when it was washed with water. Ca(OH)_2_ effectively decoupled the (Nd–Pr)_2_Fe_14_B grains and enhanced coercivity of both (Nd–Pr)_2_Fe_14_B and(Nd–Pr)_1.5_Dy_0.5_Fe_14_B. Ethanol ball milling increased the magnetic moment and BH_max_ of (Nd–Pr)_1.5_Dy_0.5_Fe_14_B (BM) but decreased the coercivity value down to 5.31 kOe (Fig. 4).

Among all three products, minimum values of magnetic moment (16.95 *μ*_B_), Mr (56.3 emu/g), and Ms (86.5 emu/g) were recorded for (Nd–Pr)_2_Fe_14_B. In contrast, Dy addition enhanced the magnetic moment (20.38 *μ*_B_), Mr (70.6 emu/g), and Ms (104.2 emu/g) values of (Nd–Pr)_1.5_Dy_0.5_Fe_14_B.

Removal of non-magnetic Ca(OH)_2_ phase further increased the magnetic moment. Hence, (Nd–Pr)_1.5_Dy_0.5_Fe_14_B (BM) exhibited the maximum values of magnetic moment (21.67 *μ*_**B**_), M_r_ (87.7 emu/g) and M_s_ (110.8 emu/g). Although Dy addition and Ca(OH)_2_ removal increased both M_r_ and M_s_ values but these factors enhanced the Mr value more as compared to the M_s_ value. This lead to the enhancement in squareness ratio, which was recorded as 0.65, 0.68 and 0.79 for (Nd–Pr)_2_Fe_14_B, (Nd–Pr)_1.5_Dy_0.5_Fe_14_B and (Nd–Pr)_1.5_Dy_0.5_Fe_14_B (BM) respectively.

## Conclusion

In a convenient chemical method (Nd–Pr)_2_Fe_14_B particle were synthesized from monazite concentrate. At first Th, Sm, U, and La were separated from monazite by roasting, solvent extraction, and leaching in HCl. Produced monazite leachate mainly consisted of chlorides of Nd and Pr. After addition of FeCl_3_, Nd and Pr and Fe chlorides were converted to Nd, Pr and Fe oxide by co-precipitation and annealing. Finally, these oxides were reduced and diffused to produce (Nd–Pr)_2_Fe_14_B. Pr in the (Nd–Pr)_2_Fe_14_B reduced its energy product because of ferromagnetic coupling with Nd and Fe. Dy addition enhanced anisotropy energy, coercivity and Mr value. Meanwhile, M_r_ value of the water washed product was reduced because of the presence of the Ca(OH)_2_, the non-magnetic by-product of the R-D. Ethanol ball milling efficiently removed the CaO, hence BH_max_ was almost tripled (from 3.9 to 11.45 MGOe). Process reported is energy-efficient, environment-friendly, low cost and time-saving.

## Supplementary Information

Supplementary Information.
