# Supplementary material for: Novel﻿ eco-friendly low cost and energy efficient synthesis of (Nd–Pr–Dy)2Fe14B magnetic powder from monazite concentrate
Source: Sci Rep. 2021 Oct 18;11:20594. doi: 10.1038/s41598-021-99464-w (PMC8523539; doi:10.1038/s41598-021-99464-w)
Supplement: Supplementary file 1 — Supplementary Information. [file 41598_2021_99464_MOESM1_ESM.docx]

Novel Eco-friendly Low Cost and Energy Efficient Synthesis of (Nd-Pr-Dy)_2_Fe_14_B Magnetic Powder from Monazite Concentrate

Syed Kamran Haider ^a,b,c^, Jin-Young Lee ^a^, Amol Uttam Pawar ^c^, Dongsoo Kim ^a,b*^, Young Soo Kang ^c^*

^a^ Convergence research center for development of mineral resources, Korea Institute of Geoscience and Mineral Resources, 124, Gwahakro, Yuseonggu, Daejeon, 34132, Korea.

^b^ Powder & Ceramics Division, Korea Institute of Materials Science, 797, Changwondaero, Seongsangu, Changwon, Gyeongnam, 51508, Korea.

^c^ Department of Chemistry, Sogang University, 35, Baekbeomro, Mapogu, Seoul, 04107, Korea.

*Corresponding authors

Email: yskang@sogang.ac.kr

KEYWORDS: Monazite, (RE)_2_Fe_14_B, Leaching, Co-precipitation, BH_max_

# Experimental Process.

Monazite consecrate was ground to the size of 44-500 μm and was mixed in water in such a way that a slurry having density of 100 g/L was produced. In order to remove U and Th, slurry was roasted in H_2_SO_4_ (6 N) at 220 ^o^C for 90 minutes. Acid to slurry weight ratio was kept as 1:3. Sulfates of U and Th were separated from RE_2_(SO_4_)_3_ (RE= Rare earth) by the solvent extraction process reported by Schrötterová et al.^1^ To remove La and Sm , sulfates of La, Sm and RE were converted to the hydroxides by precipitating with NaOH. Hydroxides precipitates were leached in HCl, then chlorides of La and Sm were separated from the solution by solvent extraction method reported by Jyothi et al.^2^ Produced the leachate solution mainly consisted of chlorides of Nd and Pr. For the sake of convenience, in this work, this solution is named as monazite leachate.

After adding the FeCl_3_.6H_2_O (and DyCl_3_.6H_2_O) into the monazite leachate, mixture was co-precipitated with NaOH. 3.5 M NaOH solution was added drop-by-drop to raise the pH of the solution up to 13 to convert all the RE and Fe chlorides to hydroxide. Obtained hydroxides of Pr, Nd, (Dy) and Fe were washed twice with DI water and ethanol, then dried at 80 ^o^C for 6 hrs. Annealing at 700 ^O^C for 30 minutes converted the RE and Fe hydroxide precipitates to the oxides. Oxides particles obtained after annealing were mixed with boric acid and CaH_2_ in a glove box and then pressed into the pellet form. For the synthesis of Nd_2_Fe_14_B by reduction diffusion method, conventional ratio for Nd:Fe:B is 15:77:8. In this work, for the synthesis of (Nd-Pr)_2_Fe_14_B Nd:Pr:Fe:B ratio was kept as 11:4:77:8 (Nd:Pr ratio is fixed as 3.83:1 in monazite leachate). For the synthesis of (Nd-Pr)_1.5_Dy_0.5_Fe_14_B, Nd:Dy:Pr:Fe:B ratio was kept as 8.35:2.9:3.75:77:8. CaH_2_: oxide weight ratio was kept as 1:1. The pellet was reduced and diffused in a tube furnace by heating at 1000 °C for 3 h. The product was washed with water again and again to remove CaO, then rinsed twice with acetone and stored in the inert environment. Flow chart of experimental process in comparison with the regular physical process method for the synthesis of Nd_2_Fe_14_B is provided as Fig. 1. Fig. 2 describes the byproduct produced (in our work and regular physical method) and their environmental impact comparatively.

# How Pr effects the structural and magnetic properties when added in Nd_2_Fe_14_B:

# To study the effect of Pr in (Nd-Pr)_2_Fe_14_B, in the separate experiments, Nd_2_Fe_14_B and Nd_1.5_Pr_0.5_Fe_14_B were synthesized (Figure S-1). Comparison of their (Nd_2_Fe_14_B and Nd_1.5_Pr_0.5_Fe_14_B) structural and magnetic was performed. The atomic ratio of Nd:Fe:B for Nd_2_Fe_14_B was kept as 15:77:8. In order to get Nd_1.5_Pr_0.5_Fe_14_B, Nd:RE:Fe:B ratio was kept as such a way that 25% of Nd was substituted with Pr.

Both the Nd_2_Fe_14_B and Nd_1.5_Pr_0.5_Fe_14_B show JCPSD #36-1296 as main phase with additional peaks corresponding to the Nd rich phase (Fig. S-2). The peaks position for Nd_2_Fe_14_B and Nd_1.5_Pr_0.5_Fe_14_B are same because their atomic radii are very close. In order to calculate the lattice parameters, d-spacing values were calculated from the XRD patterns with the help of Bragg's Law. h,k and l values were determined from Nd_2_Fe_14_B JCPSD #36-1296. Finally, “c” and “a” values for table 1 were calculated by the following equation for tetragonal crystal lattice.

$$\frac{1}{d^{2}}=\frac{h^{2}+k^{2}}{a^{2}}+l^{2}/c^{2}$$

| Particles | a | c | c/a |
| --- | --- | --- | --- |
| Nd_2_Fe_14_B | 8.80 | 12.54 | 1.425 |
| Nd_1.5_Pr_0.5_Fe_14_B | 8.80 | 12.56 | 1.427 |

Table. 1. Lattice parameters of “a” and “c” for the Nd_2_Fe_14_B and of Nd_1.5_Pr_0.5_Fe_14_B particles.

Pr substitution showed a slight enhancement in the crystal parameters (Table 1). This slight enhancement is because of Pr radius which is slightly larger as compared to the Nd. Figure S-3 shows the comparison of the morphology and particle size. Pr substitution reduced the particle size. This is maybe due to the creation of new nucleation points during the R-D process. Fig. 4-S shows the homogeneous distribution of Nd, Pr, Fe in the Nd_1.5_Pr_0.5_Fe_14_B particles.

The measured hysteresis curves for the Nd_2_Fe_14_B and Nd_1.5_Pr_0.5_Fe_14_B are shown in Figure S-5. Magnetic properties of Nd_1.5_Pr_0.5_Fe_14_B have been strongly affected by their ferromagnetic coupling with Fe in Nd_1.5_Pr_0.5_Fe_14_B crystal lattice. Magnetic moments (in *μB*, obtained from M_S_) of Nd_2_Fe_14_B and Nd_1.5_Pr_0.5_Fe_14_B, were determined as 25.50 and 26.32 *μB*. Energy product of Nd_2_Fe_14_B and Nd_1.5_Pr_0.5_Fe_14_B, was recorded as 39.7 and 33.8 KJ/m^3^. Coercivity values of Nd_2_Fe_14_B, and Nd_1.5_Pr_0.5_Fe_14_B, were determined as 4.70, and 4.00, kOe. Hence 12% reduction in the coercivity was noticed when Pr substituted the Nd. Mr values for Nd_2_Fe_14_B and Nd_1.5_Pr_0.5_Fe_14_B were recorded as 67 and 64 emu/g respectively. All the magnetic properties discussed above were determined from the hysteresis loop (Fig. S-5). Conclusively, Pr substitution in Nd_2_Fe_14_B, reduces the energy product, coercivity and M_r_ values.

**Optimization of the annealing temperature of the hydroxide annealing :**

RE and Fe oxides were also produced by annealing at 800, 900 and 1000 and 1100 ^o^C to optimize the process. By annealing at higher temperature XRD peaks of both Fe_2_O_3_ and NdFeO_3_ became sharper and their intensity also increased (Fig. S-7). Hence annealing at higher temperature increased the crystallinity as expected. But increased crystallinity of oxides had almost no effect on the magnetic properties of (Nd-Pr)_2_Fe_14_B produced from them. Hence, 700 ^o^C was opted as the optimum annealing temperature for the synthesis of oxides because it required low thermal energy.

**Figures and figure captions**


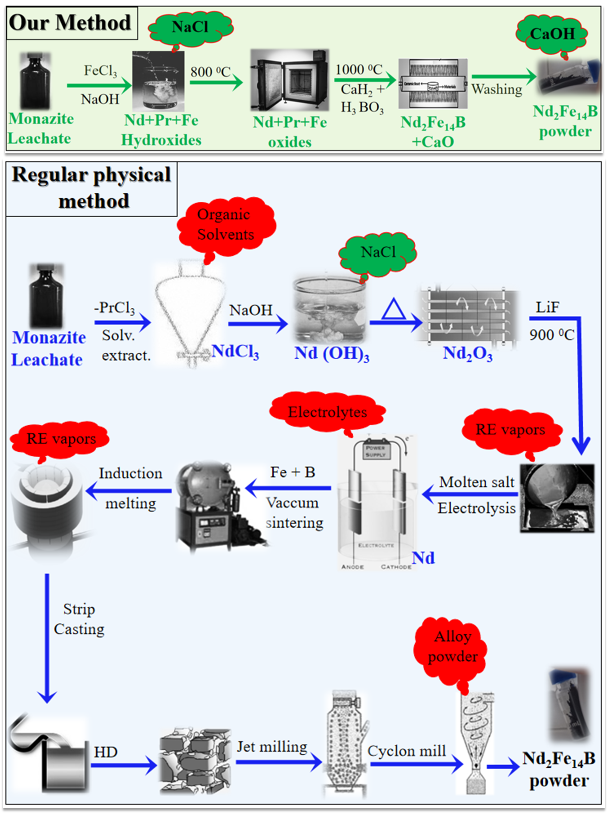


Figure S-1. (a) Flow chart of our experimental process compared with the regular commercial process showing the byproducts produced during the process. The green cloud shows harmless byproducts and red clouds indicate the harmful pollutants, produced as byproducts.


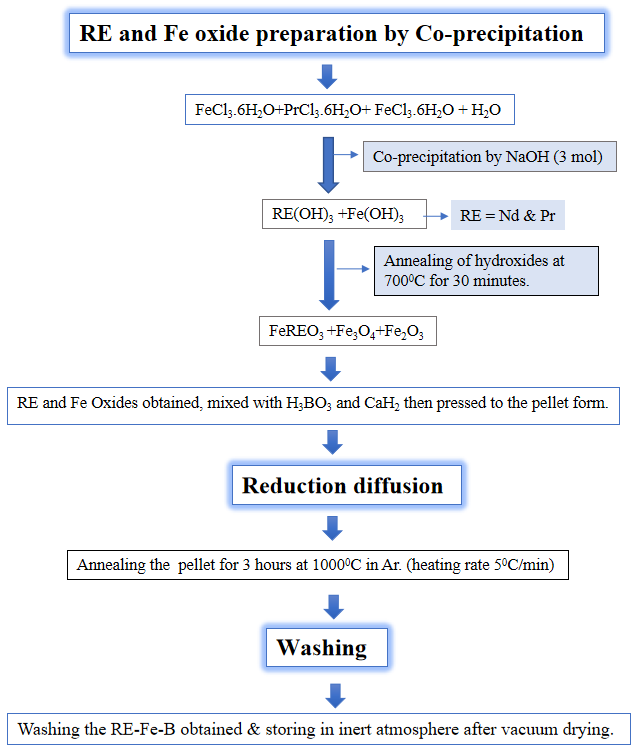


Fig. S- 2**.** Schematic illustration of the process for the synthesis of Nd_2_Fe_14_B and Nd_1.5_Pr_0.5_Fe_14_B particles.


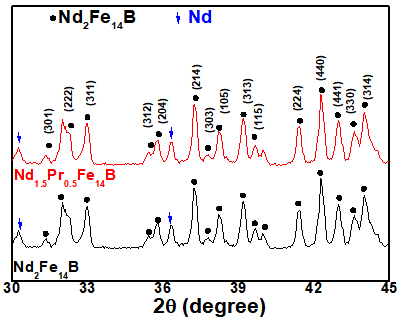


Fig. S-3. XRD patterns of Nd_2_Fe_14_B and Nd_1.5_Pr_0.5_Fe_14_B particles.


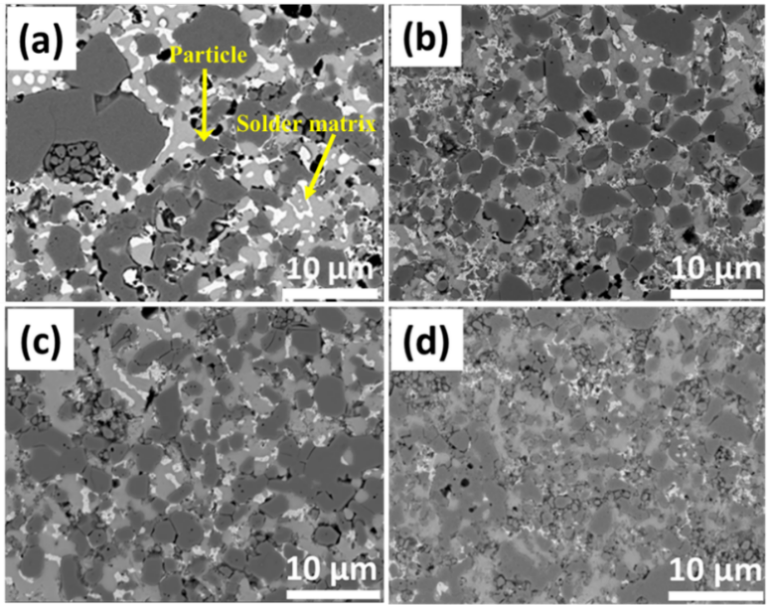


Fig. S-4. BSE-SEM images of (a) Nd_2_Fe_14_B (b) Nd_1.5_Pr_0.5_Fe_14_B


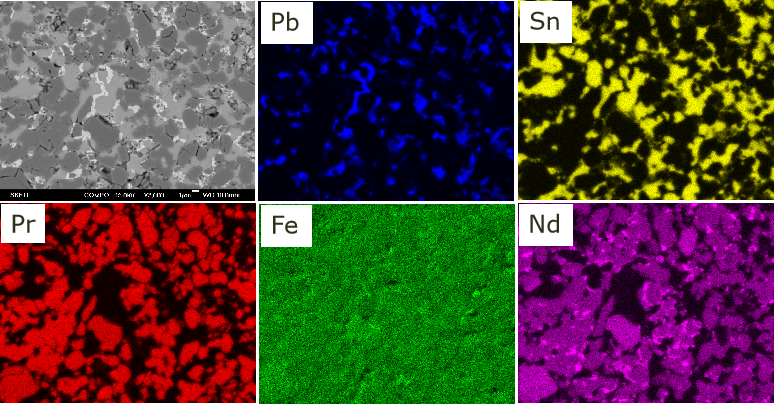


Fig. S-5. SEM-EDS images of Nd_1.5_Pr_0.5_Fe_14_B (Pb and Sn are from solder).


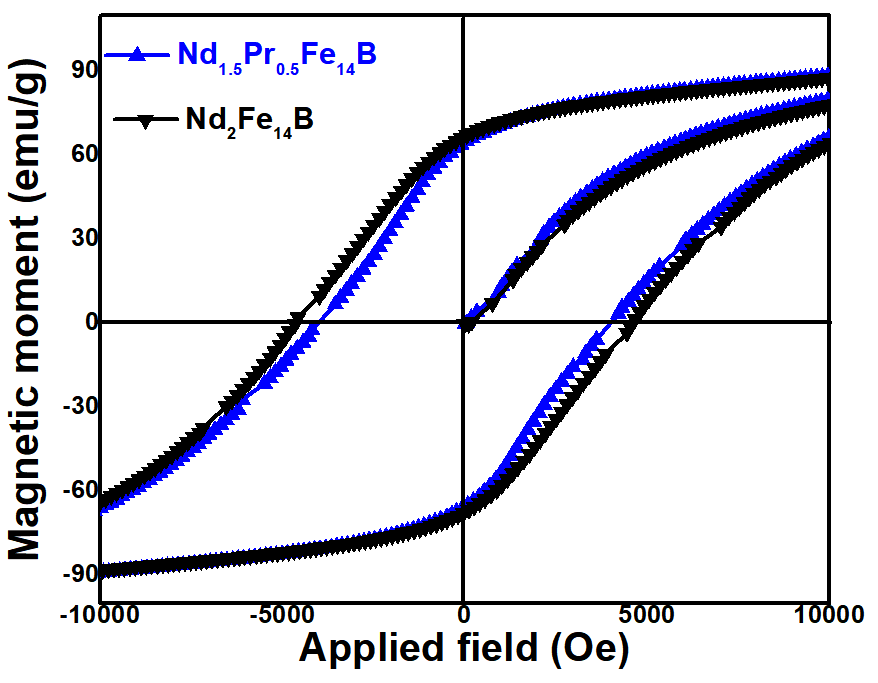


Fig. S-6. M-H curve for Nd_2_Fe_14_B, and Nd_1.5_Pr_0.5_Fe_14_B.


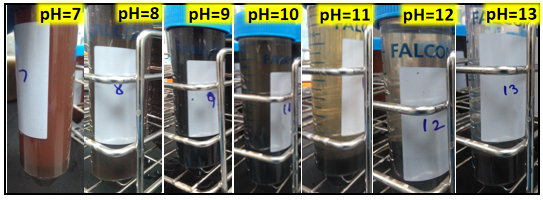


Fig. S-7. Hydroxide precipitates prepared after the co-precipitation at different pH.


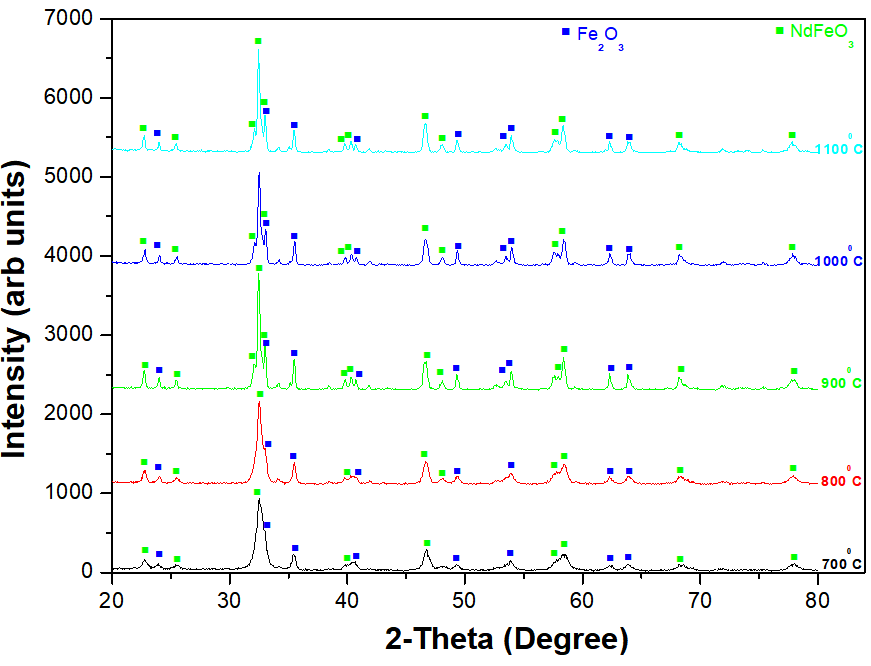


Fig. S-8. RE and Fe hydroxide precipitates annealed at 700, 800, 900, 1000 and 1100 ^o^C.


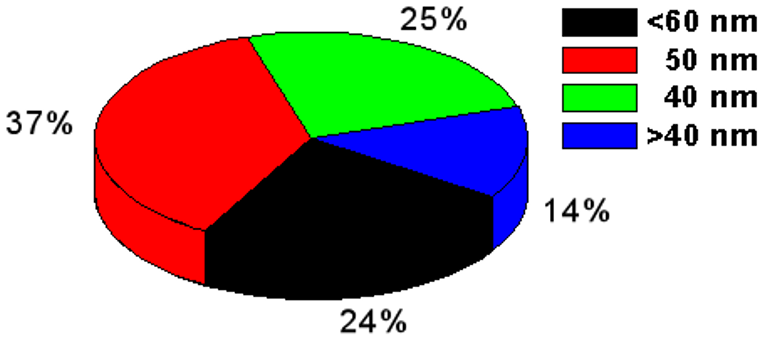


Figure S-9 Particle size distribution of RE and Fe oxides.

Fig. S-10. Percentage yield of RE and Fe oxide produced at various pH.


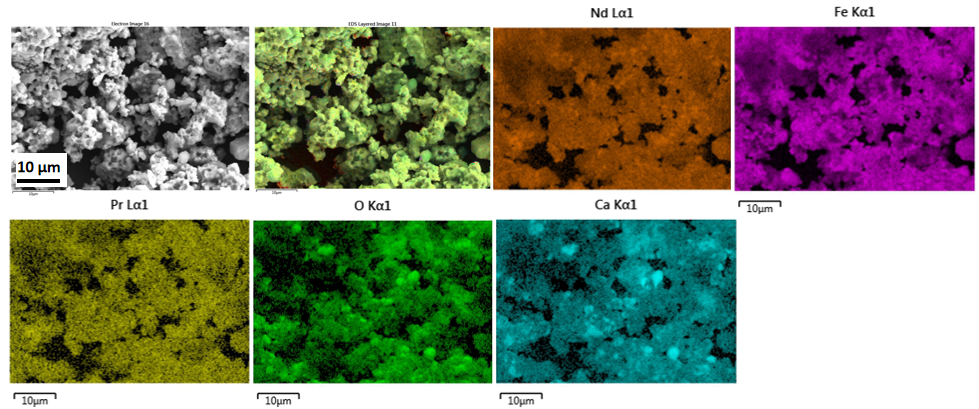


Figure S- 11. SEM-EDS of (Nd-Pr)_2_Fe_14_B.


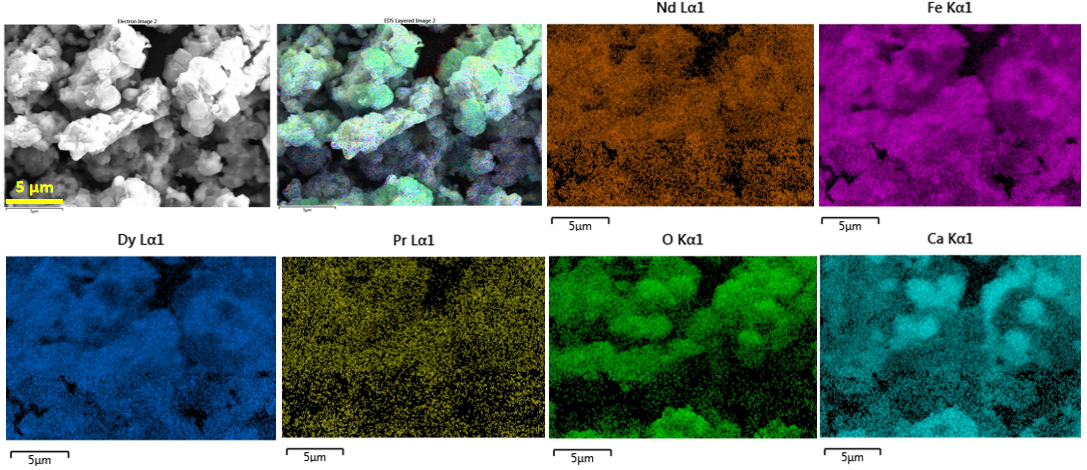


Figure S-12. SEM-EDS of (Nd-Pr)_1.5_Dy_0.5_Fe_14_B.


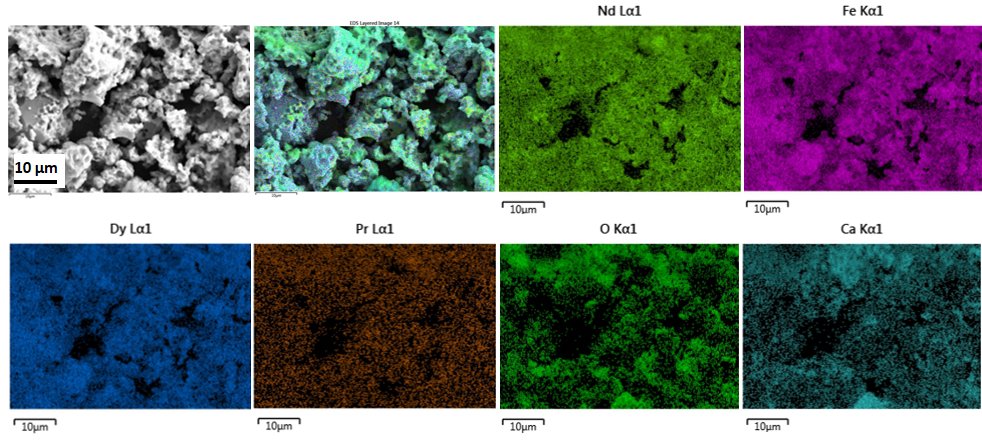


Fig. S-13. SEM-EDS of (Nd-Pr)_1.5_Dy_0.5_Fe_14_B (BM).

**Refernces’**

[(1) Schrötterová](https://www.tandfonline.com/author/Schr%C3%B6tterov%C3%A1,+D), D., [Nekovář](https://www.tandfonline.com/author/Nekov%C3%A1%C5%99,+P), P. & [Mrnka](https://www.tandfonline.com/author/Mrnka,+M), M. Extractive separation of uranium and zirconium sulfates by amines. *J. Solv. Extrac. Ion Exch.* **10** 231-241. <https://doi.org/10.1080/07366299208918102> (1992).

(2) Jyothi, R. K., Kim, H. R., Kim, J. S., Chung, K. W. & Lee, J. Y. Diluents Role in Extraction and Possible Separation of Light Rare Earth Elements from Chloride Solutions by using Cyanex 272 used as an Extractant. *Kor. J. Met. Mater.* 10, 763-771.  <https://doi.org/10.3365/KJMM.2018.56.10.763> (2018).
